# Serum neurofilament light for detecting disease activity in individual patients in multiple sclerosis: A 48-week prospective single-center study

**DOI:** 10.1177/13524585241237388

**Published:** 2024-03-13

**Authors:** M Johnsson, YT Stenberg, HH Farman, K Blennow, H Zetterberg, C Malmeström, S Sandgren, I Rosenstein, J Lycke, M Axelsson, L Novakova

**Affiliations:** Department of Clinical Neuroscience, Institute of Neuroscience and Physiology, Sahlgrenska Academy, University of Gothenburg, Gothenburg, Sweden; Sahlgrenska University Hospital, Gothenburg, Sweden; Department of Neurology, Sahlgrenska University Hospital and Region Västra Götaland, Gothenburg, Sweden; Institute of Medicine, University of Gothenburg, Gothenburg, Sweden; Clinical Neurochemistry Laboratory, Sahlgrenska University Hospital, Mölndal, Sweden; Clinical Neurochemistry Laboratory, Sahlgrenska University Hospital, Mölndal, Sweden; Department of Psychiatry and Neurochemistry, Institute of Neuroscience and Physiology, Sahlgrenska Academy, University of Gothenburg, Mölndal, Sweden; Clinical Neurochemistry Laboratory, Sahlgrenska University Hospital, Mölndal, Sweden; Department of Psychiatry and Neurochemistry, Institute of Neuroscience and Physiology, Sahlgrenska Academy, University of Gothenburg, Mölndal, Sweden; UK Dementia Research Institute, University College London, London, UK; Hong Kong Center for Neurodegenerative Diseases, Hong Kong, China; Wisconsin Alzheimer’s Disease Research Center, University of Wisconsin School of Medicine and Public Health, University of Wisconsin-Madison, Madison, WI, USA; Department of Clinical Neuroscience, Institute of Neuroscience and Physiology, Sahlgrenska Academy, University of Gothenburg, Gothenburg, Sweden; Sahlgrenska University Hospital, Gothenburg, Sweden; Department of Neurology, Sahlgrenska University Hospital and Region Västra Götaland, Gothenburg, Sweden; Laboratory for Clinical Immunology, Department of Neurology, Sahlgrenska University Hospital, Gothenburg, Sweden; Department of Clinical Neuroscience, Institute of Neuroscience and Physiology, Sahlgrenska Academy, University of Gothenburg, Gothenburg, Sweden; Sahlgrenska University Hospital, Gothenburg, Sweden; Department of Neurology, Sahlgrenska University Hospital and Region Västra Götaland, Gothenburg, Sweden; Department of Clinical Neuroscience, Institute of Neuroscience and Physiology, Sahlgrenska Academy, University of Gothenburg, Gothenburg, Sweden; Sahlgrenska University Hospital, Gothenburg, Sweden; Department of Neurology, Sahlgrenska University Hospital and Region Västra Götaland, Gothenburg, Sweden; Department of Clinical Neuroscience, Institute of Neuroscience and Physiology, Sahlgrenska Academy, University of Gothenburg, Gothenburg, Sweden; Sahlgrenska University Hospital, Gothenburg, Sweden; Department of Neurology, Sahlgrenska University Hospital and Region Västra Götaland, Gothenburg, Sweden; Department of Clinical Neuroscience, Institute of Neuroscience and Physiology, Sahlgrenska Academy, University of Gothenburg, Gothenburg, Sweden; Sahlgrenska University Hospital, Gothenburg, Sweden; Department of Neurology, Sahlgrenska University Hospital and Region Västra Götaland, Gothenburg, Sweden; Department of Clinical Neuroscience, Institute of Neuroscience and Physiology, Sahlgrenska Academy, University of Gothenburg, Gothenburg, Sweden; Sahlgrenska University Hospital, Gothenburg, Sweden; Department of Neurology, Sahlgrenska University Hospital and Region Västra Götaland, Gothenburg, Sweden

**Keywords:** Multiple sclerosis, neurofilament light, disease activity, biomarkers, prospective study

## Abstract

**Background:**

Serum neurofilament light (sNfL) reflects neuroaxonal damage and is now used as an outcome in treatment trials of relapsing-remitting multiple sclerosis (RRMS). However, the diagnostic properties of sNfL for monitoring disease activity in individual patients warrant further investigations.

**Method:**

Patients with suspected relapse and/or contrast-enhancing lesions (CELs) were consecutively included and performed magnetic resonance imaging (MRI) of the brain at baseline and weeks 28 and 48. Serum was obtained at baseline and 2, 4, 8, 16, 24, and 48 weeks. Neurofilament light concentration was measured using Single molecule array technology.

**Results:**

We included 44 patients, 40 with RRMS and 4 with clinically isolated syndrome. The median sNfL level peaked at 2 weeks post-baseline (14.6 ng/L, interquartile range (IQR); 9.3–31.6) and reached nadir at 48 weeks (9.1 ng/L, IQR; 5.5–15.0), equivalent to the median sNfL of controls (9.1 ng/L, IQR; 7.4–12). A baseline *Z*-score of more than 1.1 (area under the curve; 0.78, *p* < 0.0001) had a sensitivity of 81% and specificity of 70% to detect disease activity.

**Conclusion:**

One out of five patients with relapse and/or CELs did not change significantly in post-baseline sNfL levels. The utility of repeated sNfL measurements to monitor disease activity is complementary rather than a substitute for clinical and MRI measures.

## Introduction

Neurofilament light (NfL) protein is a biomarker of axonal damage and neurodegeneration. In multiple sclerosis (MS), it mainly reflects disease activity but may also predict disease severity^1^ and response to treatment.^2–4^ Thus, high NfL concentrations in cerebrospinal fluid (CSF) and serum are associated with relapses, lesion formation on magnetic resonance imaging (MRI), and disease worsening.^5–7^ Over recent years, serum NfL (sNfL) measurements have been investigated extensively, and sNfL has become an outcome measure in several clinical trials.^8,9^ However, the utility of monitoring sNfL changes in individual patients warrants further investigation. Although age and body mass index (BMI) adjustments have been applied to absolute sNfL reference values,^10,11^ and fixed cut-offs for pathology have been suggested,^10,12–14^ sNfL has not yet become a tool for individual monitoring of relapsing-remitting multiple sclerosis (RRMS). There is a need for standardization of the sNfL assay and for prospective studies with a high frequency of repeated sampling.^15^ We have previously investigated the range of sNfL levels during natalizumab treatment in patients with no evidence of disease activity (NEDA-3).^16^ The current study aimed to evaluate the utility of sNfL for monitoring individual patients with active RRMS.

## Materials and methods

### Study population and sampling

**Figure 1.**
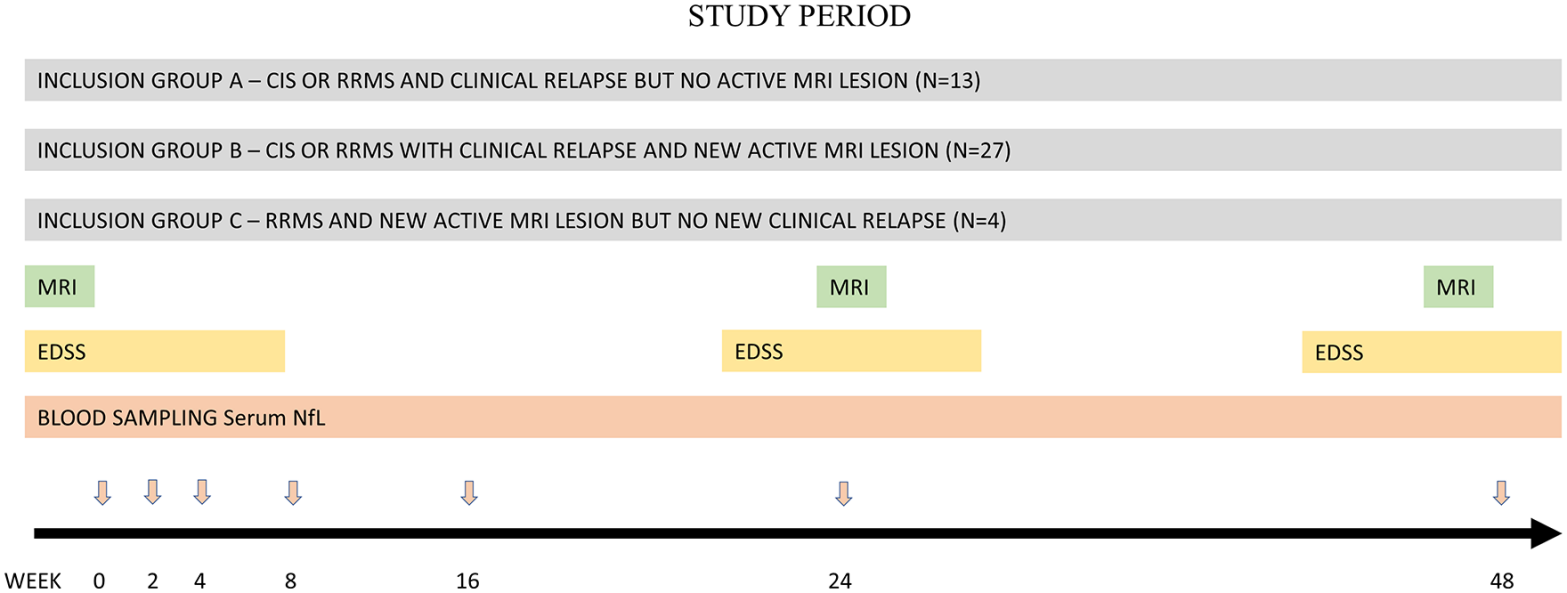
The study timeline. The black arrow indicate the number of weeks. The red arrows indicate study visits. CIS: clinically isolated syndrome; EDSS: Expanded Disability Status Scale; NfL: neurofilament light; RRMS: relapsing-remitting multiple sclerosis; MRI: magnetic resonance imaging.

This was a prospective single-center study at the MS center, Sahlgrenska University Hospital in Gothenburg, Sweden. Inclusion criteria were RRMS^17^ or clinically isolated syndrome (CIS)^18^ with a current relapse with or without one or more contrast-enhancing lesions (CELs) on MRI, or patients with RRMS with CELs without symptoms of clinical relapse. They could be treated with disease-modifying therapies (DMTs) or untreated. The baseline was defined as the first serum sampling after relapse or MRI with CEL. Exclusion criteria were other neurological diseases. Patients were consecutively included between September 2017 and January 2021. A relapse was defined as a demyelinating event with neurological disturbance lasting more than 24 hours without an alternative explanation.^19^ At the physician’s discretion, a relapse was treated with high-dose methylprednisolone 1000 mg intravenously (i.v.) for 3 days. Patients underwent clinical assessments with Expanded Disability Status Scale (EDSS)^20^ and MRI brain scans at baseline, 24, and 48 weeks. MRI was done according to a standard protocol for MS, with T1- and T2-weighted and fluid-attenuated inversion recovery sequences. A standard dose of i.v. gadolinium was applied, followed by T1-weighted imaging. Spinal MRI was done in patients with symptoms suggestive of possible myelitis, or in patients with CIS or onset of RRMS. Serum was collected at weeks 0, 2, 4, 8, 16, 24, and 48, to determine sNfL concentrations. The study timeline is presented in Figure 1.

Natalizumab-treated RRMS patients (*N* = 66) from a previous study^16^ served as controls. They had repeated sNfL measurements for 12 months (median number of measurements; 10, interquartile range (IQR); 4–10). All achieved NEDA-3, defined as a lack of relapse, no new or enlarging lesions detected on MRIs, and no significant progression during the study period.^21^

### Biomarker analysis

sNfL concentration was measured using the Single molecule array NF-light™ Advantage Kit on an HD-X Analyzer (Quanterix, Billerica, Massachusetts), as previously described.^22^ The intra-assay and inter-assay coefficients of variance (CVs) were below 10%.

For age- and BMI-adjusted normative values and sNfL concentration comparisons between groups, *Z*-scores were derived from the online application created by Benkert et al.^10^ The *Z*-score value describes how an individual patient’s sNfL concentration is related to the age- and BMI-adjusted mean level in a large group of healthy controls, and is measured in terms of standard deviations from the mean. When BMI data were missing (*n* = 5 in controls, and *n* = 15 in the study cohort), a value of 25 was used as proposed by the application.

Because the study cohort was younger than the control group, and age is a confounding factor, we only included patients under the age of 50 in calculations pertaining to baseline raw absolute sNfL concentrations.

### Statistics

Statistical analysis was done using SPSS Statistics software version 27, GraphPad Prism 9.4, and Microsoft Excel 365. A visual inspection of the data and the Shapiro–Wilk normality test showed a non-normal age distribution and sNfL data. Hence, non-parametric tests were used. The Mann–Whitney *U* test corrected for ties was applied for unpaired data, and the Wilcoxon signed-rank test corrected for ties was used for paired data. Spearman’s rank-order correlation coefficient corrected for ties was applied to determine the monotonic relations between NfL and clinical and demographic variables. The receiver operating characteristic (ROC) curve was used for classification in sensitivity and specificity calculation. Points closest to the left upper corner were chosen as cut-off values. Logistic regression was performed to determine how different sNfL variables (predictors, i.e. biomarkers) affected a patient’s odds ratio (OR) for having inflammatory disease activity (being part of the study cohort vs. controls). A *p*-value of ⩽0.05 was considered statistically significant.

### Ethical standards

All patients and controls participated voluntarily, and informed consent was obtained after providing oral and written information. The study conformed to the Code of Ethics of the World Medical Association (Declaration of Helsinki). The Regional Ethics Review Board in Gothenburg, Sweden approved the study (Dnr 1133-16).

## Results

Out of 56 patients, 8 participants did not fulfill the inclusion criteria due to symptoms or disorders that were not suggestive of CIS or RRMS (neuromyelitis optica spectrum disorder, atypical white matter disease, meningioma, secondary progressive MS, Charcot–Marie–Tooth neuropathy, Ehlers–Danlos syndrome, spinal nerve injury, fever illness). Four participants dropped out from the study after inclusion, resulting in a study cohort of 44 participants at baseline: 40 RRMS and 4 CIS. Inclusion subgroups were: 13 patients with a relapse but no CEL (30%), 27 patients with a relapse and CEL(s) (61%), and 4 patients with CEL(s) but no relapse (9%). Demographics and clinical characteristics are presented in Tables 1 and 2. Nine patients had signs of new disease activity during follow-up: three patients with new CELs, four with new T2 lesions, and two with new sensory symptoms (see Supplemental Figure 1 for sNfL trajectories).

**Table 1.** Baseline demographic and clinical characteristics

	CIS/RRMS study cohort (*N* = 44)	NEDA-3 controls (*N* = 66)	
Age, median (IQR)	35 (27–40)	45 (37–52)	*p* < 0.001
Sex, female, *N* (%)	31 (71)	54 (82)	*p* = 0.165
Years since MS diagnosis, median (IQR)	0 (0–8)	10 (6–16)	*p* < 0.001
Time in days from relapse onset or CEL detection to sampling, mean (SD)	22 (20)	NA	
Body mass index (kg/m^2^), median (IQR)	24 (21–30)^*N* = 29^	25 (19–56)^*N* = 60^	*p* = 0.746
EDSS, median (IQR)	2.0 (1.5–3.5)^*N* = 44^	2.0 (1.0–2.5)^*N* = 66^	*p* = 0.121

CIS: clinically isolated syndrome; RRMS: relapsing-remitting multiple sclerosis; NEDA-3: no evidence of disease activity; CEL: contrast-enhancing lesion; EDSS: Expanded Disability Status Scale; IQR: interquartile range; NA: not applicable; SD: standard deviation; MS: multiple sclerosis.

**Table 2.** Clinical characteristics at baseline and follow-up.

CIS/RRMS study cohort	Timepoints		
	Baseline	24 weeks	48 weeks
EDSS, median (IQR)	2.0 (1.5–3.5) ^N = 44^	2.0 (1.0–3.5) ^N = 21^	1.5 (0.0–2.0)*^p^^=^^0.076^ ^N = 42^
Relapse at timepoint, *N*	39 (89%)	2 (5%)	1 (2%)
Steroid treatment at relapse, *N* (%)	30 (68)	2 (5)	1 (2)
Cerebral MRI, *N* (%)	44 (100)	36 (82)	42 (95)
With CELs, *N* (%)	23 (52)	3 (8)	0 (0)
Number of CELs, median (IQR)	1 (1–3)	2 (1–5)	0
Spinal cord MRI, *N* (%)	23 (52)	8 (18)	6 (14)
With CELs	9 (39%)	1 (13%)	0 (0%)
Number of CELs, median (IQR)	1 (1–3)	1	0
DMT, *N* (%)	12 (27)	40 (91)	39 (89)*^p < 0.001^
Platform therapies	4	1	0
Teriflunomide	0	1	1
Fingolimod	3	2	2
Alemtuzumab	4	4	5
Cladribine	0	4	4
Ocrelizumab	0	1	1
Rituximab	1	7	10
Dimethyl fumarate	0	4	2
Natalizumab	0	16	14*^p < 0.001^
None	32 (73%)	4 (9%)	5 (11%)

CIS: clinically isolated syndrome; RRMS: relapsing-remitting multiple sclerosis; CEL: contrast-enhancing lesion; EDSS: Expanded Disability Status Scale; DMT: disease-modifying treatment; MRI: magnetic resonance imaging; IQR: interquartile range.

* Comparison to baseline.

### The influence of disease activity on individual sNfL

**Figure 2.**
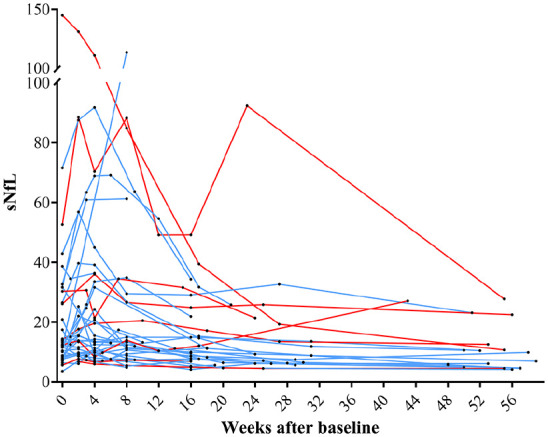
Individual sNfL concentration (ng/L) trajectories in the study cohort (*N* = 44). Patients with relapsing-remitting multiple sclerosis or clinically isolated syndrome had relapse and/or contrast-enhancing lesions at baseline. Baseline (week 0) is first serum sampling. Blue lines indicate individual patients without disease activity during follow-up, and red lines indicate individuals with disease activity during follow-up. sNfL: serum neurofilament light.

Individual sNfL concentrations for all patients in the study cohort are shown in Figure 2. The median sNfL increased from 12.4 ng/L (IQR; 8.1–26.1) at baseline to 14.6 ng/L (IQR; 9.3–31.6) 2 weeks after baseline, followed by a slow but steady decrease until the end of the study. The nadir sNfL level was reached at 48 weeks (9.1 ng/L, IQR; 5.5–15.0) equivalent to the median sNfL for controls (9.1 ng/L, IQR; 7.4–12). The concentrations of sNfL increased between baseline and week 2 (median increase 1.69, *p* = 0.001), and decreased between baseline and week 48 (median decrease –3.2, *p* = 0.003). In patients with a clinical relapse (*N* = 40), the sNfL concentration peaked at median 5.5 weeks (IQR; 4–9) after the clinical onset of relapse symptoms (see Supplemental Figure 2 for sNfL trajectories).The delay in reaching peak sNfL was not significantly different (*p* = 0.866) when excluding patients with post-baseline inflammatory disease activity, or comparing patients with or without high-dose methylprednisolone (*p* = 0.939) or highly effective DMT (not including platform therapies) at baseline (*p* = 0.819). Three participants in the study cohort had a second increase in sNfL after their primary peak in sNfL concentration post-baseline, and they were all among the nine patients with clinical or radiological evidence of new disease activity during follow-up. There was no statistically significant difference in sNfL concentrations at any timepoint between patients who, at baseline, received and did not received high-dose methylprednisolone (*p* > 0.3) or highly effective DMT (not including platform therapies; *p* > 0.1).

Individual mean sNfL was significantly higher in patients with relapse and concomitant CELs than in patients with relapse but without CELs at baseline (median difference 5.3 ng/L, *p* = 0.045), and only those with relapse and concomitant CELs had a significant change in sNfL level at follow-up (Supplemental Figure 3).

### Exploring different cut-off levels for detecting disease activity

We examined the sensitivity and specificity of different cut-off levels that were based on sNfL concentrations, individual variability of sNfL concentrations, and the *Z*-score, which takes into account age and BMI as confounding factors for sNfL (Table 3). The variability of sNfL was significantly higher in the study cohort than in stable controls, as the median individual sNfL range in patients sampled twice or more in the study cohort (*N* = 39) was 9.1 ng/L (IQR; 4.2–25.8) versus 3.6 ng/L (IQR; 2.3–4.9) in controls (*N* = 66; *p* < 0.001), and the CV was 33% and 16%, respectively (*p* < 0.001). The median baseline *Z*-score in active patients as compared to stable controls was 1.77 (IQR; 1.17–2.65) and 0.76 (IQR; 0.36–1.37, *p* < 0.001). The *Z*-score was 2.05 (IQR; 1.44–3.04) at 5.5 weeks after relapse onset. See Supplemental Figure 4 for individual sNfL ranges.

**Table 3.** Serum neurofilament light (sNfL) variables and corresponding sensitivity, specificity, and odds ratios for disease activity.

sNfL variable	AUC	Cut-off	Sensitivity	Specificity	PPV	NPV	OR (95% CI)^a^	
							Cut-off used as dichotomous factor variable	Per unit of measurement
Raw absolute sNfL at baseline^b^	0.72 (*p* = 0.0010)	>10.0 ng/L	60%	82%	77%	71%	2.8 (1.3–6.4)	1.1 (1.0–1.1)
sNfL range across the study period	0.76 (*p* = 0.0001)	>7.7 ng/L	59%	93%	82%	79%	17.5 (5.7–53.4)	1.2 (1.1–1.3)
*Z*-score at baseline^b^	0.78 (*p* < 0.0001)	>1.1	81%	70%	64%	85%	10.0 (4.0–26.0)	3.4 (2.0–5.8)
*Z*-score at week 5.5 after relapse^b,c^	0.84 (*p* < 0.0001)	>1.3	82%	71%	63%	87%	11.3 (4.3–30.0)	4.7 (2.5–8.9)
*Z*-score at baseline in patients with CELs^b^	0.81 (*p* < 0.0001)	>1.1	83%	70%	56%	90%	11.5 (3.9–34.4)	3.8 (2.1–6.8)
*Z*-score at baseline in patients with relapse but no CELs	0.72 (*p* = 0.0140)	>0.8	77%	54%	26%	94%	5.8 (1.2–28.4)	2.9 (1.2–7.1)

AUC: area under the curve; sNfL: serum neurofilament light; PPV: positive predictive value; NPV: negative predictive value; OR: odds ratio; CI: confidence interval; CEL: contrast-enhancing lesion.

If not specified otherwise, all patients in the study cohort and all controls were included in the analyses.

a Odds ratio for a patient being part of the study cohort with inflammatory disease activity versus controls.

b In controls, the individual mean sNfL was used to calculate the state variable.

c In patients with a clinical relapse (*N* = 40).

**Figure 3.**
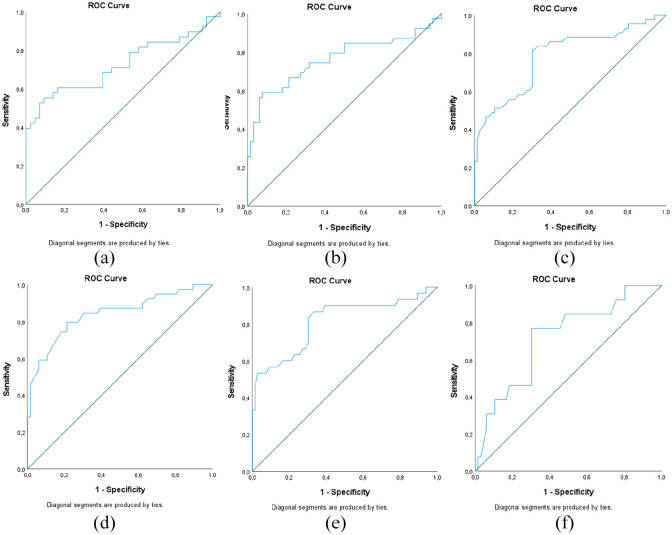
ROC curves for variables (a) absolute sNfL concentration (ng/L) at baseline in those less than 50 years old, (b) sNfL range (ng/L), (c) *Z*-score at baseline sNfL level, (d) *Z*-score at 5.5 weeks after relapse symptom onset, and *Z*-score at baseline (e) in those with MRI activity and (f) in those with relapse but no MRI activity. In controls, individual mean sNfL was used to calculate *Z*-scores in (c–f), and individual mean sNfL in controls aged less than 50 years old were used to calculate (a). ROC: receiver operating characteristic; sNfL: serum neurofilament light; MRI: magnetic resonance imaging.

We used ROC curves to find optimal cut-off values for sNfL, sNfL range, and *Z*-score (Figure 3). Based on area under the curve (AUC) values, the overall sensitivity and specificity were highest using *Z*-score. Although the specificity and OR are highest for sNfL range, it was at the expense of sensitivity. Accuracy, ORs, and sensitivity remained significant and similar after excluding patients with disease activity during follow-up. ORs and AUC values remained significant and similar in subgroups with or without high-dose methylprednisolone treatment or highly effective DMT (not including platform therapies) at baseline, except for non-significant ORs for the predictor variable sNfL range in subgroups without steroid treatment (OR; 1.11, 95% confidence interval (CI); 0.99–1.25) or with DMT at baseline (OR; 1.13, 95% CI; 0.98–1.29).

## Discussion

The aim of this study was to evaluate the utility of sNfL for monitoring individual patients with active RRMS. We assessed the sNfL kinetics in a prospective study of patients with disease activity and analyzed different sNfL variables and their corresponding sensitivity and specificity to detect disease activity.

Our data are consistent with previous studies that show a robust correlation between disease activity and sNfL concentration at the group level.^7,23–26^ Most participants in our study reached the peak concentration of sNfL 2–12 weeks (median 5.5) after the relapse onset, with a slow decline thereafter, confirming previous reports on the temporal change of NfL levels in serum and CSF that reflect disease activity.^5,12,27–29^ We showed that the median individual sNfL range in active RRMS/CIS patients was 9.1 ng/L (IQR; 4.2–25.8), that is 2.5 times higher than in patients with stable disease,^16^ and the individual sNfL CV was 33%, which was twice that of stable RRMS controls. A similar CV was found in a post hoc analysis of repeated measurements of sNfL from patients with evidence of disease activity.^12^ However, in their patients with NEDA, CV was 25% which was significantly higher than in our stable patients. This discrepancy was likely due to differences in the selection of patients and treatment. Although their patients achieved NEDA, they were treated with placebo/peginterferon beta, whereas our patients with NEDA were exposed to natalizumab treatment, a highly effective DMT.

It is important to recognize the delay between relapse onset and the peak of sNfL concentration. The temporal change in sNfL is probably due to physiological metabolic and elimination processes^5,30^ and the extent, intensity, and length of relapses and MRI lesion activity.^25^ Suspect relapses with symptoms that are not confirmed in the neurological examination or with an increase of the functional score or EDSS may be supported by an increase in sNfL. However, this assumes that there is a baseline sNfL concentration and that repeated sampling is performed. According to our data, there is still a risk of false-negative results, but the chance of detecting ongoing axonal damage increases if sNfL determinations are performed 5.5 weeks (range 2–12) after relapse onset.

The chance of having elevated sNfL is higher during periods with CELs on MRI.^28,31,32^ At the group level, patients with CELs were the only subgroup with significantly increased sNfL during follow-up in our study cohort. Similar increase of sNfL variability and temporal association between sNfL levels and MRI activity have previously been demonstrated.^33^ In the current study cohort, patients with relapse and no CELs had lower sNfL than relapsed patients with CELs, which may imply that relapses without MRI activity are primarily demyelinating and give rise to less axonal injury. However, this result should be interpreted with caution, as only 23 patients had a spinal MRI at baseline, 6 of whom were classified as having no CEL at baseline. Therefore, some patients could have had undetected spinal CEL. It is also possible that some patients had new or worsening symptoms that were not related to inflammatory activity and without CELs on MRI. Despite the limitations, we were able to calculate the sensitivity and specificity values for sNfL to detect MRI activity. Our findings suggest that sNfL has a moderate accuracy in predicting MRI activity, as sensitivity was 83% and specificity 70% (AUC; 0.81) when we used an age- and BMI-adjusted baseline sNfL variable (*Z*-score).^10^ This sensitivity level was in line with, or slightly better than results from other studies,^31,32^ although previous studies have had different designs and comparisons between studies should be done with caution. In the APLIOS ofatamumab treatment trial, 284 patients were individually profiled and sNfL trajectories were assessed. A ROC curve analysis showed that age-adjusted models with baseline or time-matched dichotomized sNfL (⩽9.1 ng/L) as predictors of MRI activity resulted in a moderate accuracy (AUC range; 0.64–0.73).^31^ Higher proportion of sub-clinical MRI activity or the use of dichotomized predictors and absolute sNfL concentrations may be reasons for a lower accuracy compared to our current study. Another recent study similarly showed that sNfL had only moderate sensitivity for detecting CELs.^32^

Only about two-thirds of patients in our cohort had increased sNfL concentrations at the peak level when compared to common upper normal reference limits,^11^ despite clinical or radiological evidence of disease activity. Therefore, we analyzed different sNfL variables to compare their accuracy to detect disease activity. We used a previous stable natalizumab-treated RRMS cohort with NEDA as controls and calculated cut-offs for the detection of disease activity. We applied the recently developed *Z*-score online application^10^ and found that age- and BMI-adjusted normal reference values are valuable tools that allow comparisons between study populations. On the other hand, the sNfL range as a parameter does not seem to correlate with age or BMI either. The advantage of sNfL range is the use of absolute raw sNfL concentrations, and our data show that a high range is very rare in stable RRMS patients. Thus, the specificity of sNfL range is higher than for *Z*-score (93% vs. 70%) but at the expense of sensitivity (59% vs. 81%). Overall, the accuracy, the clinical applicability, and the possibility to compare populations accounting for confounding factors seem to be better with the use of *Z*-score compared to sNfL range.

In this prospective study, active RRMS had repeated determinations of sNfL at intervals that are feasible for assessment of disease activity in clinical practice. We confirmed the temporal aspects of sampling, suggesting serum sampling 2–12 weeks from relapse onset or close to the appearance of CELs on MRI. We found that the *Z*-score was the most accurate measure for assessing disease activity with sNfL. Still, one out of five patients showed no increase of sNfL during disease activity. Our data do not support that sNfL can replace clinical and MRI measures for monitoring disease activity. However, in RRMS patients who achieved NEDA under highly effective DMT, sNfL concentrations were low and stable, suggesting a potential role for sNfL for long-term monitoring of inflammatory disease activity.^16^ The utility of *Z*-score from sNfL determinations in clinical practice warrants further validation.

## Supplemental Material

sj-docx-1-msj-10.1177_13524585241237388 – Supplemental material for Serum neurofilament light for detecting disease activity in individual patients in multiple sclerosis: A 48-week prospective single-center study Supplemental material, sj-docx-1-msj-10.1177_13524585241237388 for Serum neurofilament light for detecting disease activity in individual patients in multiple sclerosis: A 48-week prospective single-center study by M Johnsson, YT Stenberg, HH Farman, K Blennow, H Zetterberg, C Malmeström, S Sandgren, I Rosenstein, J Lycke, M Axelsson and L Novakova in Multiple Sclerosis Journal

sj-docx-2-msj-10.1177_13524585241237388 – Supplemental material for Serum neurofilament light for detecting disease activity in individual patients in multiple sclerosis: A 48-week prospective single-center study Supplemental material, sj-docx-2-msj-10.1177_13524585241237388 for Serum neurofilament light for detecting disease activity in individual patients in multiple sclerosis: A 48-week prospective single-center study by M Johnsson, YT Stenberg, HH Farman, K Blennow, H Zetterberg, C Malmeström, S Sandgren, I Rosenstein, J Lycke, M Axelsson and L Novakova in Multiple Sclerosis Journal

sj-docx-3-msj-10.1177_13524585241237388 – Supplemental material for Serum neurofilament light for detecting disease activity in individual patients in multiple sclerosis: A 48-week prospective single-center study Supplemental material, sj-docx-3-msj-10.1177_13524585241237388 for Serum neurofilament light for detecting disease activity in individual patients in multiple sclerosis: A 48-week prospective single-center study by M Johnsson, YT Stenberg, HH Farman, K Blennow, H Zetterberg, C Malmeström, S Sandgren, I Rosenstein, J Lycke, M Axelsson and L Novakova in Multiple Sclerosis Journal

sj-docx-4-msj-10.1177_13524585241237388 – Supplemental material for Serum neurofilament light for detecting disease activity in individual patients in multiple sclerosis: A 48-week prospective single-center study Supplemental material, sj-docx-4-msj-10.1177_13524585241237388 for Serum neurofilament light for detecting disease activity in individual patients in multiple sclerosis: A 48-week prospective single-center study by M Johnsson, YT Stenberg, HH Farman, K Blennow, H Zetterberg, C Malmeström, S Sandgren, I Rosenstein, J Lycke, M Axelsson and L Novakova in Multiple Sclerosis Journal
