# Supplementary material for: Serum neurofilament light for detecting disease activity in individual patients in multiple sclerosis: A 48-week prospective single-center study
Source: Mult Scler. 2024 Mar 13;30(6):664–73. doi: 10.1177/13524585241237388 (PMC11071597; doi:10.1177/13524585241237388)
Supplement: sj-docx-3-msj-10.1177_13524585241237388 – Supplemental material for Serum neurofilament light for detecting disease activity in individual patients in multiple sclerosis: A 48-week prospective single-center study [file sj-docx-3-msj-10.1177_13524585241237388.docx]

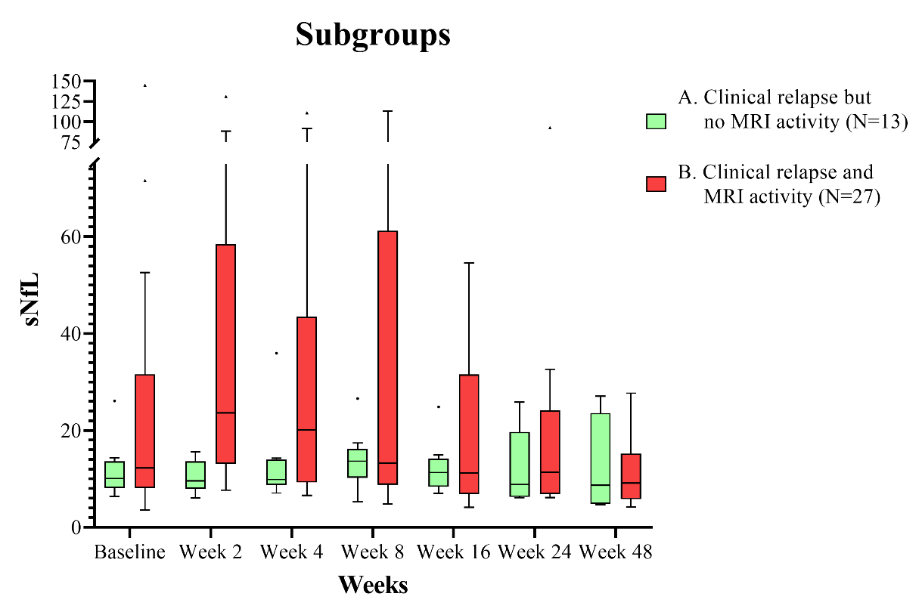


Supplementary Figure 3. Tukey boxes and whiskers depicting serum neurofilament (sNfL) levels in each subgroup in the study cohort with disease activity. Two patients in category A had T2 lesions on magnetic resonance imaging (MRI) at follow-up during the study but not at baseline. Patients with exclusively MRI activity but no clinical relapse were excluded, because this subgroup was too small for statistical comparisons (N=4).
