# Supplementary material for: Serum neurofilament light for detecting disease activity in individual patients in multiple sclerosis: A 48-week prospective single-center study
Source: Mult Scler. 2024 Mar 13;30(6):664–73. doi: 10.1177/13524585241237388 (PMC11071597; doi:10.1177/13524585241237388)
Supplement: sj-docx-4-msj-10.1177_13524585241237388 – Supplemental material for Serum neurofilament light for detecting disease activity in individual patients in multiple sclerosis: A 48-week prospective single-center study [file sj-docx-4-msj-10.1177_13524585241237388.docx]

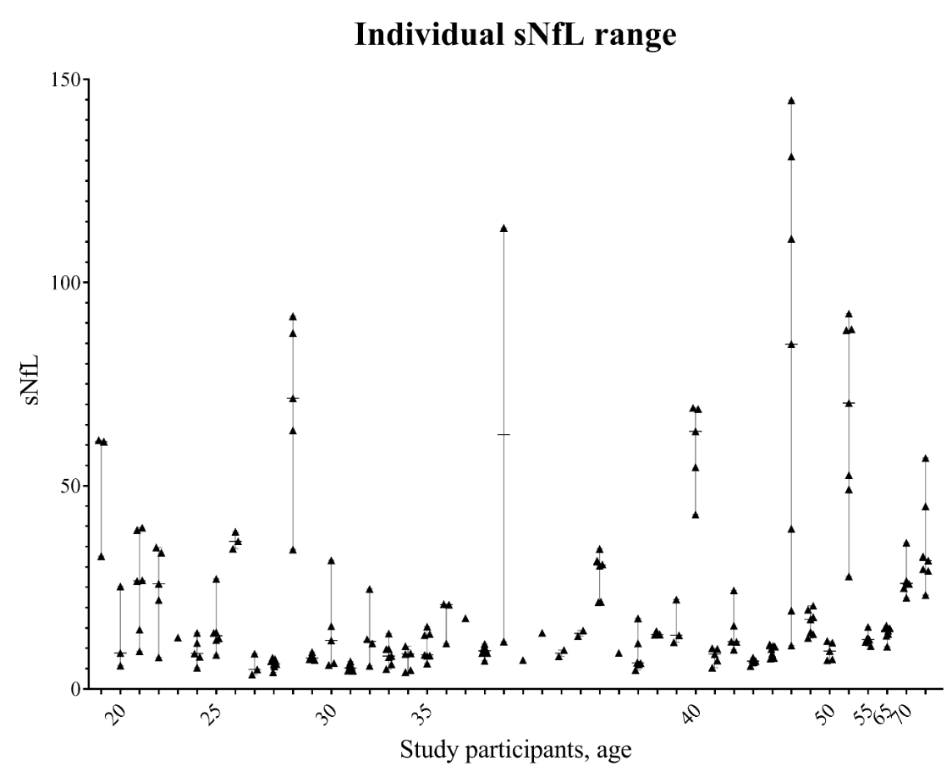


Supplementary Figure 4. Individual sNfL (ng/L) concentrations (markers) and range (vertical lines) in the study cohort (N=44), arranged according to the study participant´s age (youngest to oldest) on the x-axis.
